# Group D *Salmonella* Urinary Tract Infection in an Immunocompetent Male

**DOI:** 10.1155/2015/608632

**Published:** 2015-04-23

**Authors:** Asad Jehangir, Dilli Poudel, Shoaib Bilal Fareedy, Ahmed Salman, Anam Qureshi, Qasim Jehangir, Richard Alweis

**Affiliations:** ^1^Department of Internal Medicine, Reading Health System, Spruce Street/6th Avenue, West Reading, PA 19610, USA; ^2^King Edward Medical University, Mayo Hospital Road, Nelagumbad, Anarkali, Lahore 54000, Pakistan; ^3^Rawalpindi Medical College, Tipu Road, Rawalpindi 46000, Pakistan; ^4^Sidney Kimmel Medical College of Thomas Jefferson University, 1015 Walnut Street, Philadelphia, PA 19107, USA

## Abstract

A 62-year-old male with past medical history of benign prostatic hyperplasia presented to the emergency department with complaints of decreased urinary flow, inability to fully empty his bladder, and gross hematuria. Physical examination was unremarkable. Urinalysis revealed large amount of blood and more than 700 white blood cells suggesting a urinary tract infection. Urine culture grew group D *Salmonella* greater than 100,000 colony-forming units per mL. He was prescribed 6 weeks of trimethoprim/sulfamethoxazole and had resolution of symptoms. Retrospectively, he reported a 3-day history of watery diarrhea about a week prior to onset of urinary symptoms that was presumed to be the hematogenous source in this case. Urinary tract infection from nontyphoidal *Salmonella* (NTS) is rare and is usually associated with immunosuppression, chronic diseases, such as diabetes or structural abnormalities of the genitourinary tract. Genitourinary tract abnormalities previously reported in the literature that predispose to nontyphoidal *Salmonella* urinary tract infection include nephrolithiasis, chronic pyelonephritis, retrovesicular fistula, urethrorectal fistula, hydrocele, and post-TURP. We present an exceedingly uncommon case of 62-year-old male with group D *Salmonella* urinary tract infection predisposed by his history of benign prostatic hyperplasia.

## 1. Introduction

Urinary tract infection (UTI) from nontyphoidal* Salmonella* (NTS) is rare and is usually associated with structural abnormalities of the genitourinary tract, immunosuppression, and chronic diseases, such as diabetes. The modes of infection include direct urethral invasion, which is more common in women, or hematogenous spread from gastroenteritis. We present an unusual case of a 62-year-old male with group D* Salmonella* urinary tract infection predisposed by his history of benign prostatic hyperplasia (BPH).

## 2. Case Presentation

A 62-year-old male with past medical history of BPH presented to the emergency department with complaints of decreased urinary flow, inability to fully empty his bladder, and gross hematuria. He denied any fever, chills, nausea, vomiting, dysuria, penile discharge, lower backache, recent trauma, heavy weight lifting, or prior history of nephrolithiasis. Review of systems was negative for any diarrhea, constipation, or abdominal pain. His medications included ibuprofen as being needed for chronic right shoulder pain from rotator cuff tendinopathy and chlorpheniramine as being needed for allergic symptoms. He was not on any medications for his BPH because of very mild symptoms with concomitant low American Urological Association (AUA) Bother Score prior to this event.

On examination, he was afebrile. There was no costovertebral angle tenderness. Urinalysis revealed large amount of blood and more than 700 WBCs, suggesting UTI. Urine culture grew group D* Salmonella* greater than 100,000 colony-forming units per mL [1]. He was prescribed levofloxacin for 6 weeks for presumptive prostatic source leading to direct urethral invasion, which was changed to trimethoprim/sulfamethoxazole due to history of Achilles tendinitis and his concerns for recurrence on a fluoroquinolone antibiotic. Due to continued inability to independently urinate, a Foley catheter was placed and he was prescribed silodosin for obstructive symptoms. CT urogram with and without intravenous contrast revealed unremarkable kidneys and ureters; there was thickening of the bladder wall, likely from cystitis. The patient reported resolution of symptoms with antibiotics. Retrospectively, he recalled an episode of watery diarrhea for 3 days about a week prior to onset of urinary symptoms. This could presumably have been the hematogenous source for prostatic and subsequent urethral invasion.

## 3. Discussion

Urinary tract infection from nontyphoidal* Salmonella* was first reported in 1946 [2]. It is a rare phenomenon, accounting for 0.01 to 0.07% cases of UTIs in various studies but there has been a notable increase in the incidence of NTS infections recently [2, 3]. It is most commonly seen in infants and patients over the age of 60, like our patient [1]. In a retrospective analysis of 799 isolates of NTS from urine, serotypes of groups C1 and E were more commonly associated with UTIs, in contrast to group D* Salmonella* UTI, as described in our case [9].

The modes of urinary tract infection from NTS include hematogenous spread from gastroenteritis or contamination from fecal flora via direct urethral invasion, which is more common in women [4]. It usually manifests as typical symptoms of urinary tract infection, even though cases of asymptomatic bacteriuria have been reported. UTI from NTS is usually encountered in patients with predisposing factors, including severe immune deficiency, occult urologic problems, chronic diseases (e.g., diabetes mellitus), or exposure to reptiles, such as the common green iguana [5]. Hence an episode of NTS urinary infection must be considered as a surrogate marker of underlying predisposing factor(s), namely, heretofore unrecognized immune system suppression or compromise of genitourinary anatomy. Such patients should also be evaluated for occult diabetes or recent exposure to reptiles [4, 6]. Evaluation did not suggest any evidence of immunosuppression or diabetes in our patient. Genitourinary tract abnormalities commonly reported in the literature that predispose to NTS UTI include nephrolithiasis, chronic pyelonephritis, retrovesicular fistula, urethrorectal fistula, hydrocele, and post-TURP; our patient was conceivably predisposed to nontyphoidal* Salmonella* by his history of benign prostatic hyperplasia, as partial urinary obstruction is a major risk factor [2, 7]. Nonetheless, it is imperative to remember that these urinary infections can also occur in apparently healthy and immunocompetent individuals and there is data suggesting that the relationship of NTS UTI with genitourinary abnormalities and immunosuppression is likely an overestimation as a result of bias [8]. Thus, it is important to keep NTS in the differential of potential pathogens causing UTIs, including patients without any overt predisposing factors [4, 9]_._

NTS urine infection may be difficult to treat, but early institution of antibiotics is associated with a favorable outcome [5, 6]. Antibiotics with high intracellular concentrations such as ciprofloxacin should be used as* Salmonella* has the tendency to grow intracellularly [6]. High frequency of complications due to* Salmonella* UTI like pyelonephritis, renal insufficiency, nephrotic syndrome, nephrolithiasis, genitourinary abscesses, recurrence, and chronic bacteriuria warrants prolonged treatment, even though recurrences may still be seen [3, 4, 7].

## 4. Conclusion

Nontyphoidal* Salmonella* urinary tract infection is rare and should arouse suspicion of underlying immunosuppression or genitourinary abnormalities that can include BPH. A prolonged course of antibiotics is generally needed for NTS urinary tract infection.
